# Structures of the Insecticidal Toxin Complex Subunit XptA2 Highlight Roles for Flexible Domains

**DOI:** 10.3390/ijms241713221

**Published:** 2023-08-25

**Authors:** Cole L. Martin, David W. Chester, Christopher D. Radka, Lurong Pan, Zhengrong Yang, Rachel C. Hart, Elad M. Binshtein, Zhao Wang, Lisa Nagy, Lawrence J. DeLucas, Stephen G. Aller

**Affiliations:** 1 Department of Pharmacology & Toxicology, University of Alabama at Birmingham, Birmingham, AL 35205, USA; martin94@uab.edu (C.L.M.); docdave5@uab.edu (D.W.C.); christopher.radka@uky.edu (C.D.R.); lurong.pan@ainnocence.com (L.P.); 2 Department of Microbiology, Immunology, and Molecular Genetics, University of Kentucky, Lexington, KY 40536, USA; 3 Department of Biochemistry & Molecular Genetics, University of Alabama at Birmingham, Birmingham, AL 35205, USA; yangzw@uab.edu; 4 Department of Pathology, Microbiology & Immunology, Vanderbilt University Medical Center, Nashville, TN 37232, USA; rachel.c.hart.1@vumc.org (R.C.H.); elad.binshtein@vanderbilt.edu (E.M.B.); 5 Biochemistry & Molecular Pharmacology, Cryo-Electron Microscopy and Tomography Core, Baylor College of Medicine, Houston, TX 77030, USA; zhaow@bcm.edu; 6 Department of Mathematics, Engineering & Physical Sciences, Jefferson State Community College, Jefferson Campus, Birmingham, AL 35215, USA; lnagy@jeffersonstate.edu; 7 Predictive Oncology Inc., 200 Riverhills Business Park, Suite 250, Birmingham, AL 35242, USA; ldelucas@predictive-oncology.com

**Keywords:** TcA, toxin translocase, Cryo-EM, X-ray crystallography

## Abstract

The Toxin Complex (Tc) superfamily consists of toxin translocases that contribute to the targeting, delivery, and cytotoxicity of certain pathogenic Gram-negative bacteria. Membrane receptor targeting is driven by the A-subunit (TcA), which comprises IgG-like receptor binding domains (RBDs) at the surface. To better understand XptA2, an insect specific TcA secreted by the symbiont *X. nematophilus* from the intestine of entomopathogenic nematodes, we determined structures by X-ray crystallography and cryo-EM. Contrary to a previous report, XptA2 is pentameric. RBD-B exhibits an indentation from crystal packing that indicates loose association with the shell and a hotspot for possible receptor binding or a trigger for conformational dynamics. A two-fragment XptA2 lacking an intact linker achieved the folded pre-pore state like wild type (wt), revealing no requirement of the linker for protein folding. The linker is disordered in all structures, and we propose it plays a role in dynamics downstream of the initial pre-pore state.

## 1. Introduction

*Xenorhabdus nematophilus* is a Gram-negative bacterium that lives symbiotically with the entomopathogenic *Steinernema* nematode, which feeds on insects [1,2]. Once inside the insect host, the bacterium secrete multiple toxins and virulence factors to kill the insect while preserving the cadaver for nematode consumption [1]. A total of 15 species of *Xenorhabdus* have been discovered from various nematodes, and some of the bacterial toxins used to kill insect hosts have been characterized [1,3]. Of these are a class of multi-protein ABC toxin complexes (Tcs) that have multiple receptor binding domains on the shell of the A subunit (TcA) for recognizing and binding target cells [1].

ABC Tcs are a group of pore-forming toxins that have drawn increased attention due to their high specificity for surface specific antigens, cargo-carrying capabilities, and cytotoxic function. A deeper insight into the structure-function of Tcs could assist in the bioengineering applications that have been proposed for controlling major pests in agriculture [4,5,6], inhibiting mosquito vectors for West Nile and Zika viruses [7,8] and for medicinal biotechnology [9,10]. The more distantly related Bt-toxins currently serve as genetically engineered biopesticides in the agricultural industry [11,12,13], but insect pests have become resistant, leading to the need for alternatives such as Tcs [14,15]. Tcs also have the potential to be useful in clinical applications. Roderer et al. [10] proposed TcdA1, with 43% sequence identity to XptA2 (see Figure S1), as a universal protein translocator, and they demonstrated its ability to transport a variety of proteins across the membrane boundary. XptA2, specific to certain insects, has potential biomedical applications due to its close homology to other human pathogenic Tcs. Yet, it belongs to a phylogenetic clade of Tcs generally nonpathogenic to mammals (see Figure S2). No structural information has been obtained for any member of the XptA2 clade.

Functional studies of Tcs show that some target a broader number of species while others are very specific. The most widely studied TcA, TcdA1, has been shown to target both insect and mammalian cells [10,16,17]. The *Xenorhabdus nematophilus* TcA, XptA2, has thus far only displayed toxicity to insects (1). *Xenorhabdus* contains the loci for two distinct TcA sequences (XptA1 and XptA2), one TcB and one TcC sequence [18]. XptA1 and XptA2 share 59% sequence identity, with an amino acid length of 2538 for XptA2 and 2523 for XptA1, and isoelectric points at 5.07 and 5.09, respectively. Interestingly, XptA1 and XptA2 target different subsets of insects [19]. XptA1 displayed toxicity and morphological changes to mammalian cells in complex with B and C subunits to form the full Tc [20]. Multiple A, B, and C subunits are commonly observed in other Gram-negative bacteria that express Tcs [20]. Sergeant et al. showed that lysates containing XptA1/BC were lethal to two species of cabbage butterfly (*P. brassicae* & *P. rapae*) but not the cabbage moth (*P. xylostella)*. In contrast, XptA2/BC was lethal to the moth, non-toxic to *P. rapae* and ~100-fold less toxic to *P. brassicae* when compared to XptA1/BC [18,19]. Although XptA2 is highly similar to both XptA1 and TcdA1, XptA2 appears to exhibit the narrowest range of target specificity. A deeper understanding of the differences in binding motif structures will help identify conformational dynamics that distinguish nonpathogenic from pathogenic Tcs. Studying the functional domains will help create new constructs through protein engineering with the potential to exhibit new potencies against specific insects, serving as next-generation pesticides.

Gatsogiannis et al. [21] determined the first high-resolution structure of a broad-spectrum Tc obtained from *Photorhabdus luminescens*, TcdA1 (2516 aa and pI: 5.27). Since this discovery, the Tc from *Xenorhabdus nematophilus*, XptA1, and mammalian specific Tcs from *Yersinia* have been structurally determined [20,22,23]. Although other TcA structures have been solved, TcdA1 has had the most extensive and comprehensive analysis and will be used for comparisons throughout this manuscript. Studies show that ABC Tcs consist of 3 subunits, which facilitate cytotoxic effects on selective cells [24]. The balloon shaped TcA subunit represents ~80% of the tripartite Tc. Each TcA protomer contains 2–4 putative Receptor Binding Domains (RBDs) per protomer located on the outer shell of the TcA subunit. Each TcA protomer assembles to create an internal cavity that forms a buried translocation channel and an oligomeric surface decorated with RBDs [20]. The TcB subunit is a gated linker connecting the TcA and TcC subunits [16]. The TcC subunit contains a cytotoxic enzyme, usually an ADP ribosyltransferase or RHO GTPase, encased inside a protective cocoon. After linearization and toxin translocation into the cell, the ADP ribosyltransferase or RHO GTPase refolds upon cell entry and subsequently arrests actin polymerization or protein synthesis within the target cell, respectively [1,24].

**Figure 1:**
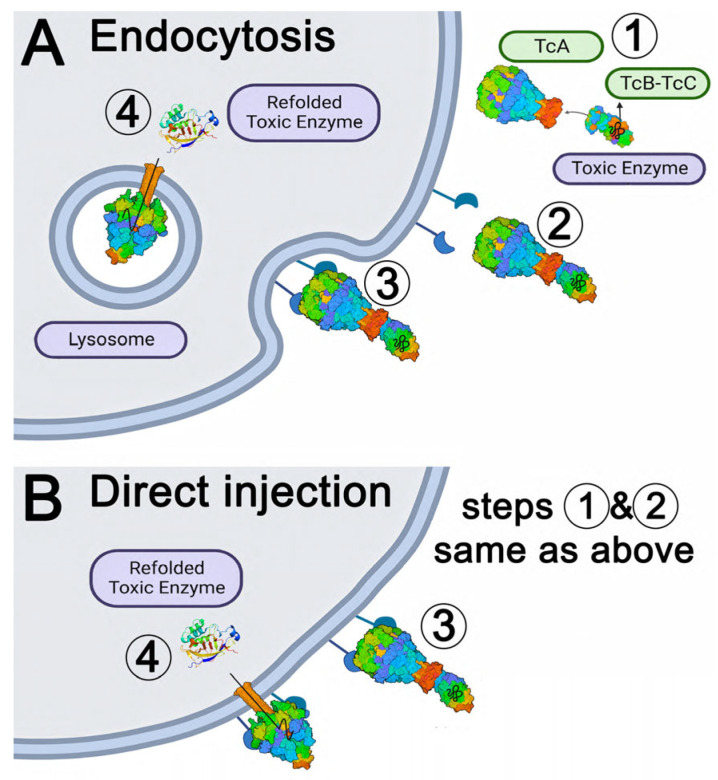
Proposed mechanisms of ABC Toxin Complex (Tc) cell recognition and cytotoxicity. (**A**) Central mechanism. Step 1: TcA and TcB-TcC subunits are secreted from bacteria (e.g., *Xenorhabdus*, *Photorhabdus*, *Yersinia*, etc., see Figure S2 for phylogenetic analysis); The structure presented here is an insect specific TcA designated XptA2 secreted by *Xenorhabdus nematophila*. Step 2: holotoxin formation by complex assembly; Step 3: recognition, binding to cell surface membrane receptor(s), endocytosis, and transport to lysosome; Step 4: pH-dependent change to pore state and injection of the toxin peptide into the cytoplasm where it folds into an enzyme that is toxic for cell division [26]. (**B**) Alternative mechanism. Steps 1 and 2 as with the central mechanism; Step 3: recognition and binding of Tcs to cell surface membrane receptors; Step 4: the high pH of the insect gut may allow the transition to the pore state at the plasma membrane and direct injection of the toxin into the cytoplasm [23].

Early studies suggest that when Tc binds its target receptor on the cell surface (11,17), the Tc is endocytosed and transported to the lysosome. Tcs exposed to the acidic pH of the lysosome undergoes a conformational change in which the outer sheath of the TcA subunit retracts over the inner translocation channel that is coupled to the TcB and TcC subunits [17,22]. A pore is formed through the lysosomal membrane when the hydrophobic residues in the translocation channel are exposed, and the cytotoxic payload within the TcC subunit (ADP ribosyltransferase or RHO GTPase) is injected into the cytoplasm. Payload cytotoxicity depends on the refolding, linearization, and translocation of the peptide within the TcC subunit across the lipid bilayer (Figure 1A). Another model for insect specific Tcs proposes that binding and translocation occur on the cell plasma membrane and is driven by the basic pH of the insect hemolymph system [23,24,25] (Figure 1B). The insect specific Tc, XptA2, has not been structurally determined, and the structural basis for its unique properties are unclear.

Here we report the high-resolution structure of the *Xenorhabdus nematophilus* TcA XptA2 by X-ray crystallography and cryo-EM, each at 3.1 Å resolution. Both structures reveal a pentameric XptA2 pre-pore state, refuting earlier claims of a tetramer [1]. A molecular comparison of the two structures identifies a dynamic RBD that may play a unique role in conformational dynamics and/or receptor binding. RBD-B emerges as a hotspot for flexibility that is either unique to XptA2 or not yet described in other TcA orthologs. Finally, a continuous ‘linker region’ that is conserved in TcA orthologs is not necessary for pre-pore folding. This is consistent with its proposed role in protein dynamics downstream of the pre-pore conformation [17]. We show a two-fragment (“2-fragment”) construct of XptA2 assembles into a pentamer, and we compare the stability of this construct with wt XptA2.

## 2. Results

### 2.1. Structure of XptA2 by XRC

**Figure 2:**
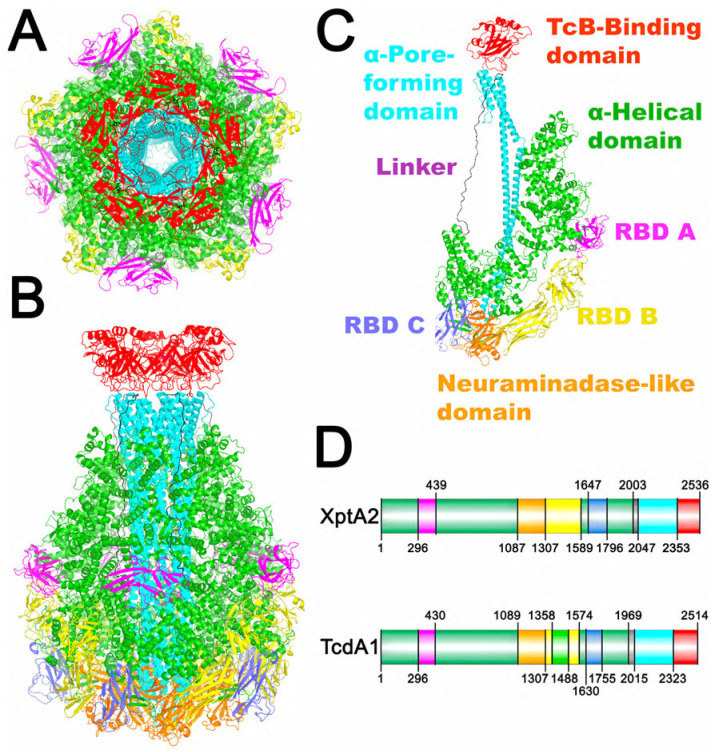
Structure of XptA2 from Xenorhabdus nematophila. (**A**) Bottom view of XptA2 colored by domain. (**B**) Side view of XptA2 colored by domain. (**C**) A single chain of XptA2 with each domain color-coded and labeled. (**D**) Domain comparison of XptA2 and TcdA1 according to the color schematic in A, B, and C.

To determine the pre-pore structure of XptA2, we utilized X-ray crystallography and single particle cryo-EM to obtain two independent structures with a final overall resolution of ~3.1 Å each. We obtained high-quality X-ray diffraction data using Se-Met incorporated XptA2 protein, but phasing power proved too weak to solve the structure, and heavy metal soaks were insufficient. We, therefore, collected single-particle cryo-EM data on native protein and calculated a ~7 Å resolution map that revealed XptA2 exhibits five-fold symmetry (C5) like other TcA orthologs [20]. The symmetry contrasted a previous report that inferred XptA2 forms a tetramer from native gel electrophoresis [1]. Pentameric symmetry is now widely accepted as the common architecture among TcA subunits of various ABC toxins, including XptA1 [20]. We then used our 7 Å map for iterative MR-SAD phasing [27] to successively locate the selenium atoms in the X-ray diffraction data, which resulted in the final electron density map to 3.1 Å resolution. De novo model building was initiated manually by placing polyalanine α-helices where they were evident. The starting model was imported into PHENIX [28], and successive rounds of phenix.model build using sequence constraints and manual editing to achieve the final model (Figure 2; R_work_ = 0.17, R_free_ = 0.24) with acceptable protein geometry (see Table S1). XptA2 forms a 17.5-nm-wide, 24-nm-long pentamer with a molecular mass of ~1.4 megadaltons (MDa). The balloon-like structure is formed from the outer shell of XptA2 that spans the central translocation channel resulting in a widening of the protein at the end of the shell. This area covers the hydrophobic residues necessary for pore formation (Leu2171-Ala2187). During the process of refinement with full non-crystallographic symmetry (NCS), we observed that a small domain of chain D was out of density (2mFo-DFc), which was confirmed by inspecting negatively (−3 σ) and positively (+3 σ) contoured mFo-DFc maps. We determined that enforcing full NCS except for constraints on residues 1363–1506 (RBD-B) produced optimal refinement of the model (see Section 4 and Figure S3). An examination of XptA2 crystal packing revealed that RBD-B of chain D is packed against RBD-A of chain E of another molecule (Figure S4). For each chain, 57 seleno-methionines from the total of 61 methionines (i.e., 93%) in the primary amino acid sequence could be identified by anomalous difference Fourier electron density (≥4σ, see Figure S5).

### 2.2. Structure of XptA2 by Single-Particle Cryo-EM

To generate a structure in the absence of crystal packing, we next solved the pre-pore structure of XptA2 using single particle cryo-EM, which yielded a map at 3.1 Å resolution (Figure S6). The cryo-EM and X-ray structures exhibit 5-fold internal symmetry comprising five protomers that assemble as a pentamer. XptA2 shares the conserved α-helical translocation channel that is less than 1 nm in diameter, housed within a balloon-shaped outer shell.

**Figure 3:**
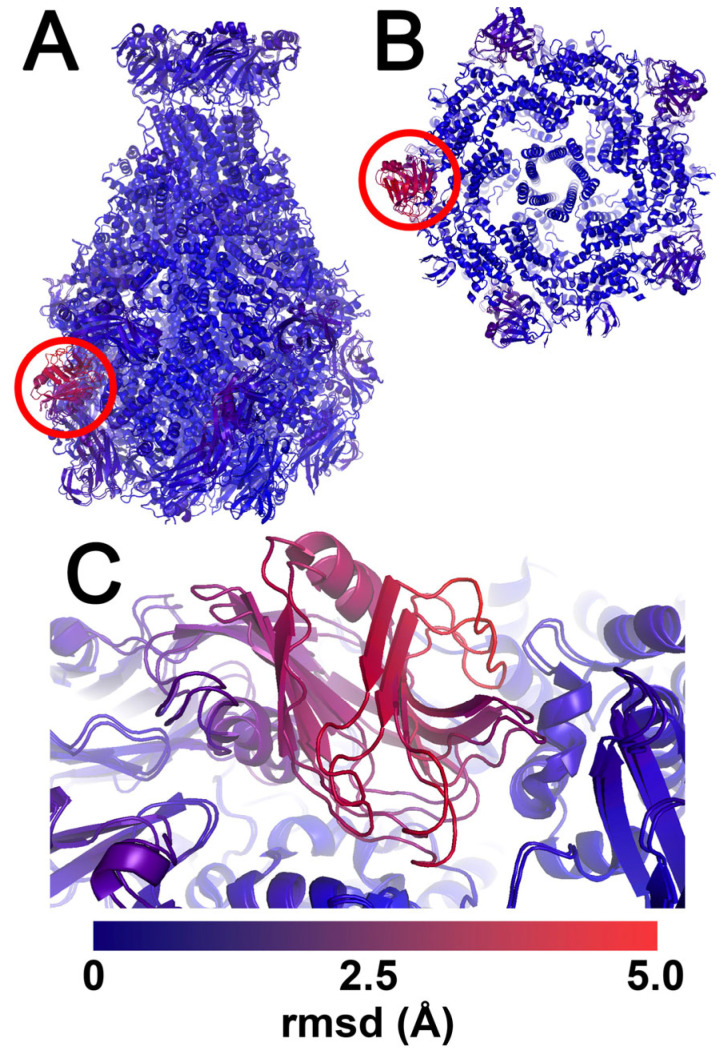
RMSD comparison of the structures of XptA2 by X-ray crystallography and Cryo-EM. (**A**) Side view of XptA2 with RBD-B (residues 1365–1508) of chain D encircled. (**B**) An end-on view of XptA2 with RBD-B (residues 1365–1508) of chain D encircled. (**C**) Zoom of XptA2 chain D shows the indentation of RBD-B due to lattice packing of this specific X-ray structure (See Figure S3 for 2mFo-DFc and cryo-EM map comparisons). RBD-B chain D flexibility is likely a property of this domain in all five pentamers and highlights a potential hotspot for conformational dynamics and/or receptor binding. The scale bar shows the color gradient of the RMSD schema used for all three panels. The panels were created within PyMOL using the Python module colorbyrmsd.py written by Shandilya, Vertrees and Holder.

Like TcdA1, two major domains per protomer constitute the outer shell of the protein that shields the central pore-forming domain from the outside environment when fully assembled [21]. One of the outer shell domains on each protomer consists mainly of α-helices, while the other is comprised mostly of β-sheets. Within these β-sheets on the outside shell of each protomer is a neuraminidase-like domain that has an extended loop oriented toward the loops of the other neuraminidase-like domains of the pentamer, thus shielding the pore-forming hydrophobic residues of the translocation channel from the outside environment. The remaining β-sheets are composed of three IgG-like receptor binding domains (RBDs) that may play a role in cell surface receptor recognition and targeting. Both structures reveal that the RBDs are like sockets in grooves with only modest contact with the main XptA2 shell. A direct comparison of the X-ray and cryo-EM structures revealed that the break in the five-fold symmetry of RBD-B of the X-ray structure results in this domain being pushed slightly inwards toward the shell due to the lattice packing (Figure 3). To our knowledge, this is the first TcA ortholog to display this type of structural flexibility for any of the RBDs in the pre-pore state specifically. Such flexibility could indicate that RBD-B is important for cell surface recognition and/or conformational change.

The RBD-B amino acid sequence is twice as long as the RBD-A or RBD-C sequences. Other orthologs have classified this area of β-sheets as two independent RBDs [17]. Close inspection of the RBD-B motif in XptA2 between amino acid residues 1307 and 1589 clearly shows two clusters of β-sheets are intertwined and not independent domains like RBD-A or RBD-C. Due to this observation, we decided to keep the nomenclature for this area of XptA2 as one continuous RBD (RBD-B) (Figure 2C,D).

### 2.3. Properties of the XptA2 Translocation Channel

**Figure 4:**
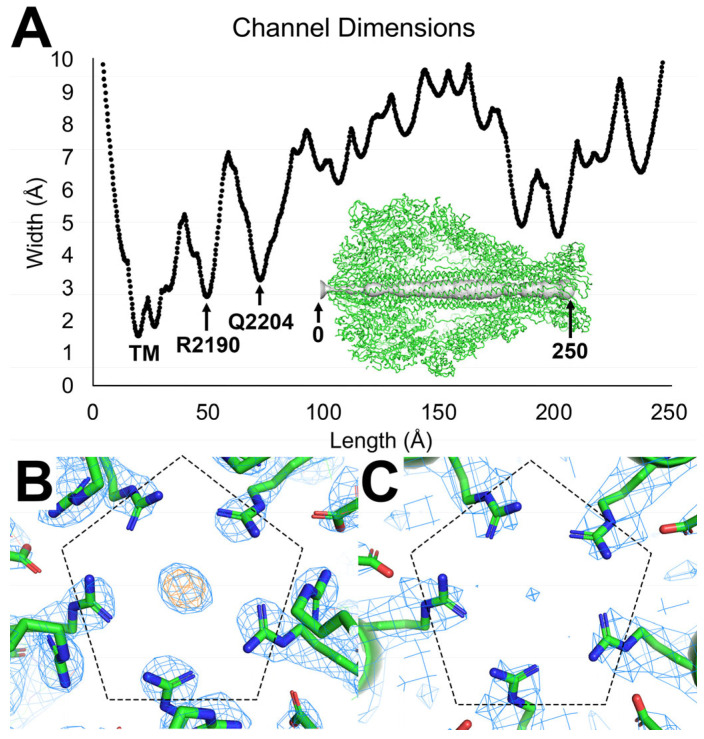
XptA2 pore dimensions and constriction analysis. (**A**) Width constrictions over the 250 Å translocation channel. The tightest dimension (1.5 Å) occurs at the transmembrane (TM) region, followed by a constriction at R2190 (~2.5 Å) and another at Q2204 (~3 Å). (**B**) Constriction of the XptA2 pore from the five-fold arrangement of R2190 side chains in the X-ray crystal structure. 2mFo-DFc map is colored in blue mesh contoured to 1.5 σ, and the mFo-DFc map is colored orange contoured to 7 σ. A strong unknown density appears at the five-fold axis. (**C**) Same view of R2190 for the cryo-EM structure as panel (**B**). Cryo-EM density is colored in blue mesh contoured to 5 σ.

The central translocation channel is formed by five extended α-helices per protomer for 25 α-helices. The shortest α-helix is located in close proximity to the C-terminal end of the protomer spanning residues Ala2337-Arg2355 and is connected to the remaining α-helices by a 23 amino acid coil (Leu2315-Ala2337). The remaining four α-helices per protomer make up the majority of the channel and are arranged in pairs running anti-parallel to each other to create a sealed inner cavity. The two α-helices leading up to the hydrophobic residues that make up the membrane piercing portion of the channel are separated by a small six amino acid coiled region (Arg2274-Lys2279) while the two α-helices running anti-parallel coming down from the membrane piercing residues are separated by a five amino acid coiled region (Glu2136-His2140). The 240 Å long pore averages ~6 Å in diameter, with the largest diameter (~10 Å) being near the B and C subunit binding sites at the C-terminal end of the protein. The smallest diameter (1.5 Å) is near the tip of the translocation channel, where the hydrophobic membrane-spanning residues are concealed (Figure 4). This hydrophobic region of the translocation channel, located at the beginning of the channel, spans 24 Å in length from residues L2171-A2187 and is important in pore formation in other TcA orthologs after Tc undergoes a conformational change into the pore state [29].

We observed Arg2190 from each protomer orients to create a ring of five inward-facing arginine residues at the second narrowest point (2.7 Å) in the channel (Figure 4B); the transmembrane region at the tip of the channel creates the narrowest point (1.5 Å). Unknown density was measured in the center of the arginine ring, apparently coordinated by the positively charged amine groups of the five arginine residues. Previous data suggests that pH change drives conformational change in ABC Tcs [29]. This hypothesis has not yet been confirmed for any Xenorhabdus ABC toxin. Although the unknown density seems to be in a functionally relevant area for TcA subunit normal physiological processes, we believe this density is not related to the pre-pore-to-pore state transition mechanism. We expect the density corresponds to a counter ion in the crystallization buffer because we do not measure density at this position in the cryo-EM structure.

### 2.4. Importance of a Continuous Linker Region for Proper Pre-Pore Folding

**Figure 5:**
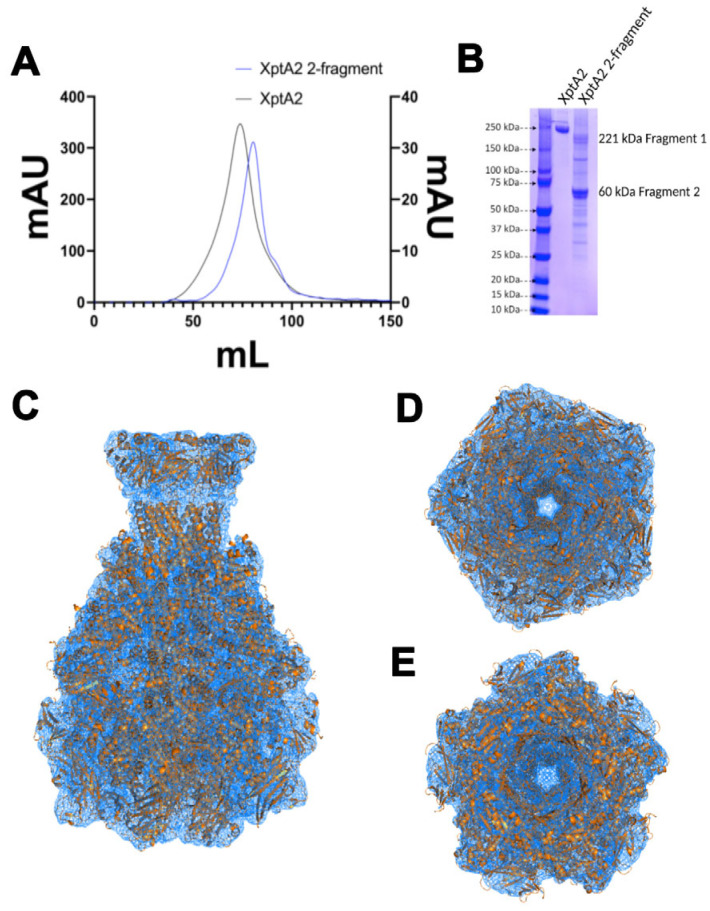
Pre-pore state of two-fragment XptA2 at 8.2 Å resolution. (**A**) Overlay of size exclusion chromatographs (SEC) of wt XptA2 and the XptA2 2-fragment construct. (**B**) SDS-PAGE comparing wtXptA2 and 2-fragment XptA2 after SEC. (**C**) Side view of the refined model for 2-fragment XptA2 (orange cartoon) fit into the experimental cryo-EM density (blue mesh). (**D**) 2-fragment XptA2 viewed from the toxin-binding face of the pentamer. (**E**) View of 2-fragment XptA2 from the transmembrane piercing face.

In other characterized TcA subunits, as well as XptA2, an unstructured stretch of ~18 amino acid residues (XptA2 residues Gln2010–Leu2027) within the “linker region” (His2003–Phe2050) is observed in the space between the outer shell and the inner translocation channel (Figure 2C). Previous studies of TcdA1 offered the conclusion that the residues that span the linker serve as an entropic spring that provides enough force to pull the outer sheath toward the rear of the shell to complete the pre-pore to pore state transition [17]. To understand whether this region containing the continuous linker is important for pre-pore folding in XptA2, we designed two expression plasmids to encode fragments of XptA2. The first fragment contained residues 1–2013, terminating near the beginning of the linker region. The second began with an artificial start codon (methionine) at the equivalent of position 2014 of the full sequence, thus leaving a zero-gap break between positions 2013 and 2014 with no peptide bond formation possible. This strategy preserves the presence of the entire linker region, albeit discontinuous. Protein purified from cells co-expressing both fragments yielded a particle with a molecular mass consistent with pentameric XptA2 by size exclusion chromatography (Figure 5A). SDS PAGE confirmed both fragments were present in the purified sample (Figure 5B).

To further ascertain whether the 2-fragment construct folded properly, we determined the structure of the pre-pore 2-fragment construct to 8.2 Å resolution using single-particle cryo-EM (Figure 5C–E and Figure S7). The XptA2 2-fragment pre-pore cryo-EM structure reveals a pentamer that is almost identical when compared to our structure of XptA2 wt (see Table S2), with a root-mean-square deviation (RMSD) of 0.019 Å for all Cα atoms. In addition to the role of the linker proposed for TcdA1 in protein dynamics [17], we conclude that an intact linker is not necessary for the proper folding or pentameric assembly of pre-pore state XptA2. This construct can be used to determine a functional role for the linker region in XptA2 conformational changes in future experiments.

**Figure 6:**
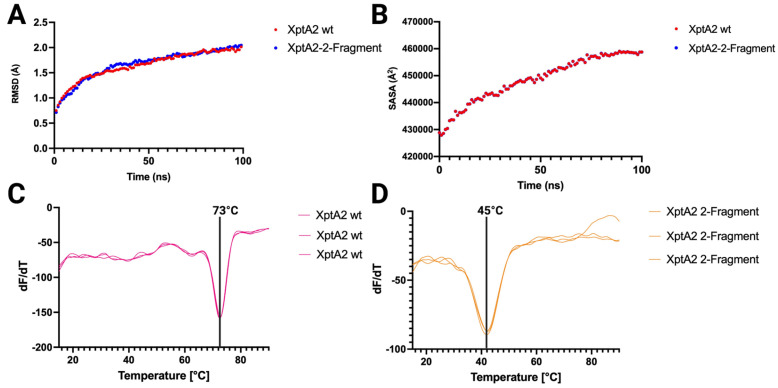
Molecular Dynamics and Differential Scanning Fluorometry of wt vs. 2-fragment XptA2. (**A**) RMSD comparison of wt XptA2 to 2-fragment XptA2 over 100 ns. (**B**) Solvent-accessible surface area (SASA) comparison of wtXptA2 to 2-fragment XptA2 over 100 ns. (**C**) Fluorescence emission at 330 nm as a function of temperature for the wt XptA2. (**D**) Fluorescence emission at 330 nm as a function of temperature for the 2-fragment XptA2.

We next used molecular dynamics (MD) simulations to compare structurally dynamic features of XptA2 with and without a continuous linker region (Figure 6). The RMSD values show XptA2 wt and XptA2 2-fragment simulations reaching a stable production phase with similar equilibrium states (Figure 6A). The measurement of the solvent-accessible solvent areas of XptA2 and the XptA2 2-fragment construct are displayed over time (Figure 6B). The 2-fragment construct displays no significant difference in solvent accessible solvent area than XptA2 wt, indicating little variability between the two data sets. This data suggests that the stability of both proteins is similar and that there are very little computationally dynamic differences between the pre-pore states of both XptA2 wt and the XptA2 2-fragment construct. The stability of both proteins was also assessed using Differential Scanning Fluorometry (DSF). The thermal unfolding of the proteins was monitored by measuring the changes in intrinsic fluorescence emission at 330 and 350 nm, mainly from the tryptophan residues, which are numerous in both proteins. Changes in tryptophan emission intensity reflect changes in environmental hydrophobicity, which decreases during unfolding as these buried residues are exposed to the aqueous solution. The DSF curves of both proteins displayed only one unfolding transition but with vastly different unfolding temperatures (Tm, the midpoint of each peak in the dF/dT plot in Figure 6C,D). The Tm of XptA2 wt is ~73 °C while the Tm of XptA2 2-fragment is ~45 °C. These data show the linker stabilizes the Tc fold but does not participate in the oligomeric assembly.

## *3.* Discussion

XptA2 has been shown to target major agricultural pests such as the tobacco budworm, *Heliothis virescens* and the corn earworm, *Heliothis zea* [1,6,18,19]. The other *Xenorhabdus nematophilus* TcA, XptA1, has been shown to target the large cabbage butterfly, *Pieris brassicae*, and the small cabbage butterfly, *Pieris rapae*; however, the differences between XptA1 and XptA2 that confer variable target specificity for the toxin complexes remain poorly understood [1]. Due to the importance of TcAs for the use of biological pesticides in large-scale crop production and the relationship between TcAs and human pathogenicity, we solved the first high-resolution structure of XptA2 through both X-ray crystallography and cryo-EM. This work allowed us to compare XptA2 to structurally similar TcAs in the field, such as TcdA1 and XptA1. We confirmed that XptA2 folds as a pentamer (Figure 2) and displays many other similar features compared to structurally characterized TcAs, such as a conserved translocation channel, a loosely ordered linker region, and multiple putative IgG-like RBDs in each protomer (Figure 2). Due to the structural and genetic homology between the XptA2 and TcdA1, it is reasonable to hypothesize that the mechanism of both TcAs is similar. XptA2 contains a 250-Å translocation channel composed of ~306 residues lining the inner surface that are highly conserved between the species (Figure S1).

Similarly, the hydrophobic residues on the end of the translocation channel are ~24 Å long for optimal membrane permeation once the pre-pore-to-pore state transition occurs. The linker region is proposed to be the driving force for conformational change in TcdA1. The XptA2 linker is disordered and “loose” in the pre-pore state between the translocation channel and the outer shell, like the TcdA1 linker. Additionally, the IgG-like domains on the outer shell of each protomer are in the optimal position for membrane receptor interaction. We, therefore, conclude that XptA2 has a similar mechanism of action as TcdA1.

Interestingly, our X-ray and cryo-EM structures allowed distinct conclusions about the differences between TcA orthologs and between the XptA2 structures. One difference between the two XptA2 structures observed was a slight shift in the positioning of RBD-B (residue Ala1310–Lys1591) in chain D. When compared to the cryo-EM structure enforced by 5-fold symmetry, RBD-B on the X-ray structure exhibits a slight indentation with respect to the outer shell. This subtle difference between the position of RBD-B on all chains is likely due to lattice packing during crystal formation. It allows visualization of certain degrees of flexibility in the domain. This dynamic movement indicates a “pop-socket” interaction between RBD-B and the XptA2 scaffold. We surmise the button-press flexibility of the putative XptA2 RBDs enhances the toxin’s binding capacity and efficiency to target insect gut cell surface receptors before translocation. Also, the flexibility of RBD-B makes it a potential candidate for genetic modification for potential bioengineering applications.

We also observed an unknown ion density within the translocation channel of the XptA2 crystal structure at the second most constricted region of the channel (2.8 Å in width). This region contains five inward-facing arginine residues, and this density is observed directly in the center of all five residues. The unknown density most likely represents a counter-ion deriving from the crystallization conditions due to the absence of this ion in the cryo-EM structure. Interestingly, the arginine ring at position 2190 in XptA2 has similar counterparts in other TcA proteins, such as TcdA1, where the authors suggest that the charges in this region likely affect channel conductivity [20]. This region could be one of many electrostatic regions that need to be destabilized, resulting in the pre-pore-to-pore state transition at a high pH.

A few of the TcAs that have been characterized display diverging trigger mechanisms that drive the binary transition from the pre-pore to pore state, which suggests that changes in pH may not be the only driving force for conformational change in Tc orthologs [17,23]. A high-resolution structure of *Photorhabdus luminescens* Tc, TcdA1, was determined in the pre-pore and pore states, revealing considerable detail about the trigger mechanism that penetrates the membrane and allows injection of the toxin into cells [17,21]. The authors report that a linker region is a tight spring to provide energy for conversion to the pore state. To assess the importance of a continuous linker region for full pentameric assembly into the pre-pore state, we have made mutations rendering the linker discontinuous in XptA2. After expressing and purifying the 2-fragment XptA2 construct, our cryo-EM data demonstrates that an intact linker region is not necessary for the proper folding of XptA2 to achieve the pre-pore state. This 2-fragment XptA2 construct sets the stage for future experiments aimed at determining the role of the linker in the conformational change of XptA2. Our MD simulations suggest the pre-pore stability of the 2-fragment construct is similar to XptA2 wt under normal conditions; however, our DSF data exhibits different Tms when comparing XptA2 wt to the 2-fragment construct. For XptA2 wt, the main thermal unfolding event occurs at ~73 °C, indicating a highly stable protein consistent with its pentameric assembly. For the XptA2 2-fragment construct, the Tm was significantly lowered to ~45 °C. This indicates that the 2-fragment construct is only marginally stable at elevated temperatures. However, at room temperature or lower, the protein is still completely folded and exhibits similar structural stability as the wt protein (Figure 5A,B).

In this work, we structurally characterized a TcA from *Xenorhabdus nematophilus*, XptA2, using X-ray crystallography and cryo-EM. We also identified common functional motifs that informed the function and mechanism of the protein. This work confirms XptA2 pre-pore characteristics similar to other TcAs, such as the pentameric symmetry, and sets the groundwork for future mechanistic studies of conformational change. Future XptA2 research will explore the utility of the full Tc in the agricultural industry and the exploration for TcAs to be used in a clinical setting for potential bioengineering applications. Thus it is important to understand their structures and function across orthologs.

## 4. Materials and Methods

### 4.1. Constructs

The full-length XptA2 wt DNA (WP_041979038.1.) was subcloned into the pET24a vector (Novagen) with a C-terminal 6× Histidine tag. The XptA2 2-fragment construct was synthesized by expressing the XptA2 wt sequence into two plasmids. The first 6039 base pairs were expressed in the kanamycin (Kan) selectable pET24a (Novagen) plasmid, and the last 549 base pairs were expressed in the ampicillin (Amp) selectable pET22b plasmid (Novagen) after adding an artificial start codon (methionine) at the beginning of the sequence. These plasmids were co-transformed into BL21(DE3) competent cells (Thermo Fisher Scientific, Waltham, MA, USA) and colonies were selected from plates containing both Amp and Kan to ensure that both plasmids were transformed into the cells.

### 4.2. Protein Expression and Purification

For X-ray crystallography, seleno-methionine (SeMet) labeled XptA2 was expressed in *Pseudomonas fluorescens* from the American Type Culture Collection (ATCC strain 13525) and purified according to Madduri et al. [30]. For cryo-EM, unlabeled recombinant wild type (wt) XptA2 and the “2-fragment” construct were expressed in *Escherichia coli* BL21(DE3) competent cells (ThermoFisher Scientific, Waltham, MA, USA). Cells were grown in Luria Broth (LB) to an OD_600_ of 0.6, chilled to 16 °C, and induced with 1 mM isopropyl-β-D-thiogalactoside (IPTG) (ThermoFisher Scientific) for 18 h. The cells were then harvested and resuspended in lysis buffer containing 20 mM Tris pH 7.5, 100 mM NaCl, 20 mM Imidazole, 0.05% Sodium Azide, and protease inhibitor cocktail (Sigma-Aldrich, Burlington, MA, USA) and lysed using a cell disruptor (Constant Cell Disruption Systems, Daventry, UK) at 15 psi. After centrifugation at 38,400× *g* for 45 min to remove cell debris, the supernatant was collected and applied to a pre-equilibrated (20 mM Tris pH 7.5, 100 mM NaCl, 20 mM Imidazole, 0.05% Sodium Azide, and protease inhibitor cocktail) nickel affinity AP-2 column (Waters) at 4 °C, eluting in 20 mM Tris pH 7.5, 100 mM NaCl, 300 mM Imidazole, 0.05% Sodium Azide, and protease inhibitor cocktail. The eluate was concentrated with a 100 kDa cutoff concentrator (Millipore, Burlington, MA, USA) and ultracentrifuged at 217,000× *g* for 30 min at 4 °C in a TLA 120.2 (Beckman Coulter, Brea, CA, USA) rotor. The supernatant was applied to a HiPrep 16/60 Sephacryl S-400 HR column pre-equilibrated with 100 mM NaCl and 10 mM Tris pH7.5. SDS PAGE was utilized to analyze XptA2 purity within the eluted fractions and the pure fractions were concentrated using a 100 kDa cutoff concentrator (Millipore) and used for further structural analyses.

### 4.3. X-ray Crystallography

Crystallization trials conducted on the same day as the purification of SeMet-labeled XptA2 protein were critical to achieving reproducible diffraction quality sample, and unfrozen protein was always required. Protein was subjected to crystallization screens purchased from Hampton Research (“Crystal Screen 1”, “Crystal Screen 2”, and “Natrix”), Emerald Biosciences (“Wizard 1” and “Wizard 2”) and an in-house incomplete factorial screen. All trials were done in sitting-drop format using a Rigaku Phoenix crystallization robot. Redundant trays were incubated at 4 °C, 15 °C and 22 °C. The best crystals were optimized by manual setup and were grown in 0.1 M HEPES, pH 7.5, 0.2 M NaCl and 16–20% PEG-3000 in capillaries or the Cryschem sitting drop format (Hampton Research, Aliso Viejo, CA, USA, HR3-159). Further information regarding the SeMet–labeling protocol can be found here [30]. High-resolution diffraction data were collected at APS beamline 22-ID. X-ray energies were tuned to the selenium K-edge (0.97932 Å) as determined by the MAD scan. Diffraction data was collected using a Mar225 detector set at a distance of 400 mm. Data were merged and scaled using HKL-2000 (HKL, Inc., Charlottesville, VA, USA). Phasing the structure using the Selenium anomalous signal (SAD) proved unsuccessful, prompting attempts to derivatize XptA2 using heavy metals. Soaks with Ta6Br12, platinum and tungsten clusters exhibited some labeling due to anomalous differences in Fourier electron density using a refined mo. However, diffraction quality was always substantially reduced, and low phasing power prevented the phasing of an experimental map. We, therefore, sought to achieve experimental electron density for the protein using cryo-EM. Further information regarding special NCS refinement can be found in Figure S3.

### 4.4. Single Particle Cryo-Electron Microscopy

#### 4.4.1. Cryo-EM Grid Preparation

For the initial 7.2 Å map of pre-pore wt XptA2, unfrozen protein (at 6 mg/mL in 16 mM tris, 40 mM NaCl, 20% glycerol, pH 7.5) was shipped to the cryo-EM core operated by the Baylor University College of Medicine (BCM) for sample processing. Protein was diluted in glycerol-free tris buffer to 1.0 mg/mL at BCM. Grids were frozen using a Vitrobot Mark IV (ThermoFisher Scientific, Waltham, MA, USA) using blot time of 4 sec, force of 2, and plunged into liquid ethane. Grids were screened for adequate particle density and ice thickness using a JEOL2010F microscope.

For the high-resolution pre-pore structure of XptA2 and the structure of 2-fragment XptA2, cryo-EM grids were prepared using a Vitrobot Mark IV (ThermoFisher Scientific, Waltham, MA, USA). Four microliters of XptA2 wt at 1.5 mg/mL for each sample (10 mM Tris, 100 mM NaCl, pH 7.5) were applied to a glow-discharged Quantifoil holey carbon grid (R2/2), blotted for 4 s at a blot force of 4 before plunge freezing into liquid ethane. Grids were stored in liquid nitrogen before shipping for screening and data collection.

#### 4.4.2. Cryo-EM Imaging

For the initial 7.2 Å map of pre-pore wt XptA2, data were collected on a JEOL3200FSC (Jeol, Tokyo, Japan) at the BCM ATC core with a Gatan K2 Summit direct electron detector (Gatan, Inc., Pleasanton, CA, USA) in super-resolution mode. Seven hundred sixty-eight (768) movies (@ 50 frames per movie) were collected with defocus ranging from 0.5 µm to 4 µm and a total dose of 80 e^−^/A^2^.

For high-resolution pre-pore wt XptA2, data were collected using the Vanderbilt University Technai T30 Polara (FEI) microscope (FEI, Hillsboro, OR, USA) equipped with Gatan K2 Summit (Gatan, Inc., Pleasanton, CA, USA) in counting mode. A total of 1993 movies (@ 50 frames per movie) were collected with defocus ranging from 0.8 µm to 2.5 µm and a total dose of 75 e^−^/A^2^.

For the 2-fragment XptA2, data were collected using a Technai F20 microscope (FEI, Hillsboro, OR, USA) equipped with a Gatan K3 detector in super-resolution mode housed in the cryo-EM core at the University of Alabama at Birmingham. A total of 26 movies (@ 40 frames per movie) were collected with defocus ranging from 0.9 µm to 2.5 µm and a total dose of 80 e^−^/A^2^.

#### 4.4.3. Cryo-EM Data Processing

For the initial 7.2 Å map of pre-pore wt XptA2, the first 25 frames of each movie were motion-corrected using MOTIONCOR2 [31] with no dose weighting and 3× binning for a final pixel size of 1.32 Å. The contrast transfer function (CTF) of the corrected sums was determined with CTFFIND4.1.5 [32], and 740 images were selected with CTFs ≤ 10 Å. The best image’s CTF had a resolution of 4.0 Å, and the average resolution was 5.6 Å. Manual picking of 1008 particles from 10 micrographs using RELION3.0.8 [33], particle extraction and 2D classification resulted in all ten classes resembling credible views of TcA, including strong 5-fold symmetry evident in end-on views. Autopicking from all 26 micrographs using the ten 2D references resulted in 94,000 particles subjected to two more rounds of classification and sorting. A final of 8 selected 2D classes (generated from 30,888 particles) was subjected to ab initio model building in C5 symmetry. The 30,888 particles were Autorefined and polished to a final 7.2 Å resolution. This map was used to solve the high-resolution X-ray structure using molecular replacement [27] and single-wavelength anomalous difference (SAD) Fourier [34] (MR-SAD) as described above.

For the high-resolution pre-pore structure of wt XptA2, each movie was motion-corrected using MOTIONCOR2 [31] with dose weighting and no additional binning for a pixel size of 1.268 Å. CTF parameters were fitted using CTFFIND4.1.5 [32], and 1605 images were selected with the highest fitted resolution beyond 5 Å. The best image CTF signal was 3.11 Å, and the average resolution was 4.10 Å. Using RELION-3.1.2 [35], a randomly chosen set of 165 micrographs were used to Autopick particles using the Laplacian function [33] with diameters 220 Å and 320 Å. Particles were extracted, scaled to 3.8 Å/px, and 2D classified in two rounds to generate seven classes used for reference-based Autopicking on all 1605 micrographs. 316,858 particles were extracted to high-resolution (1.268 Å/px) and classified to produce 15 classes used for ab initio model generation in C5. Two rounds of 3D classification produced a final of 198,591 particles that were subjected to 3D Autorefinement, CTF refinement, Bayesian polishing and postprocessing to yield the final 3.02 Å map (for a pictorial representation of the workflow, see Figure S5). A slight stretching due to a calibration error of the microscope was noticed when comparing the final X-ray and cryo-EM models, which produced the final calibrated pixel size of the Polara at 1.244 Å.

For the 2-fragment XptA2, each movie was motion-corrected using MOTIONCOR2 [31] with no dose weighting and 4× binning for a final pixel size of 2.6 Å. CTFs were determined with CTFFIND4.1.5 [32], and resolutions ranged between 6–9 Å for all 26 micrographs. Micrographs exhibited good contrast and clearly revealed a dominant set of particles in the pre-pore state. Using RELION-3.0.8 [33], 95 particles representing orthogonal side- and top-views were picked from 5 micrographs, sufficient to generate two 2D classes for Autopicking. One thousand six hundred fifty-three (1653) particles were selected for ab initio model building in C5 symmetry, and this model was used to Autopick and select a final of 3477 particles for Refinement and Bayesian polishing to yield the final ~8.2 Å map (for a pictorial representation of the workflow, see Figure S6).

### 4.5. Molecular Dynamics Simulations

All-atom molecular dynamics simulations were performed on both protein species with and without a continuous linker. All MD simulations utilized the CHARMM22 forcefield with the CMAP correction of all-atom protein forcefield46 at 12 Å isotropically at equilibrium. Free ions with a concentration of 0.15 M NaCl and 0.002 M of MgCl_2_ were added to mimic a physiological ionic condition and to neutralize the total system charge. All atomistic MD simulations were performed using NAMD2.9. Conventional MD simulations were performed for all models to examine the thermodynamic stability and potential structural transformation for each starting conformation. The simulations were performed at isothermal–isobaric ensemble (NPT ensemble) at a body temperature of 310 K and 1-atmosphere pressure. The constant temperature was maintained with Langevin dynamics using a damping coefficient of 1 ps^−1^. Constant pressure (1.01325 bar) was maintained using Nosé-Hoover Langevin piston pressure control with the Langevin piston period of 200 fs and Langevin Piston decay of 100 fs. The time steps were set to 2 fs for the entire simulation, and trajectories at every 5000 steps (10 ps) were saved for analysis. Each system was first subjected to conjugate gradient minimization for 10,000 steps (20 ps). Then, various groups of harmonic constraints were applied for a multi-step simulation to achieve the final equilibrium. The system temperature was increased from 0 to 310 K at a rate of 1 K/ps with the constraints (force constant k = 1 kcal/mol/Å^2^) on the protein. We then maintained the same constraints for another 5 ns simulation to achieve an equilibrium of water. For the last step, a starting constraint (force constant k = 5 kcal/mol/Å^2^) was applied to the protein backbone and ligands only and a gradual removal of the constraints at a speed of 0.5 kcal/mol/Å^2^ per 500 ps. To observe potential conformational changes under conventional unbiased MD simulations, a 100 ns production phase was performed without any constraint on each model. Structural analyses were based on the STRIDE algorithm. The electrostatic potential calculation used APBS 11 plugin within PyMOL. PyMOL and VMD were used for modeling and molecular image creation.

### 4.6. Differential Scanning Fluorimetry (DSF)

Thermal unfolding temperature (T_m_) and the aggregation temperature (T_agg_) of XptA2 wt and XptA2 2-fragment were determined using the Prometheus NT.48 NanoDSF instrument (NanoTemper Technologies, LLC, South San Francisco, CA, USA) with 48 capillary chambers. Each sample was excited at 290 nm, and emission was detected simultaneously at 330 and 350 nm. Protein aggregation was monitored using the built-in back-reflection optics. The samples were heated from 15 to 95 °C using a constant heating rate of 2 °C/min. The first derivative of the fluorescence signal at 330 nm versus temperature produced a bell-shaped thermal unfolding peak. The midpoint of the peak corresponded to the apparent T_m_. The fluorescence signal at 350 nm versus temperature produced a similar unfolding peak with identical Tm values.

## Data Availability

Data is available on request from the authors. The data that support the findings of this study are available from the corresponding author [S.G.A.], upon reasonable request.

## Supplementary Materials

The following supporting information can be downloaded at: https://www.mdpi.com/article/10.3390/ijms241713221/s1. References [36,37,38,39] are cited in the Supplementary Materials.
