# Supplementary material for: Structures of the Insecticidal Toxin Complex Subunit XptA2 Highlight Roles for Flexible Domains
Source: Int J Mol Sci. 2023 Aug 25;24(17):13221. doi: 10.3390/ijms241713221 (PMC10487846; doi:10.3390/ijms241713221)
Supplement: Supplementary file 1 [file ijms-24-13221-s001.zip › ijms-2562291-supplementary.pdf]

Supplementary Material

```
TcdA1       ---MNESVKEIPDVLKSQCGFNCLTDISHSSFNEFRQQVSEHLSWSETHDLYHDAQQAQK 57
XptA2       MYSTAVLLNKISP--TRDGQTMTLADLQYLSFSELRKIFDDQLSWGEARHLYHETIEQKK 58
XptA1       MIKVNELLDKINR--KRSGDTLLLTNISFMSFSEFRHRTSGTLTWRETDFLYQQAHQESK 58
                   :.:*    . .     *:::.. **.*:*:  .  *:* *:  **::: : .*

TcdA1       DNRLYEARILKRANPQLQNAVHLAILAPNAELIGYNNQFSGRASQYVAPGTVSSMFSPAA 117
XptA2       NNRLLEARIFTRANPQLSGAIRLGIE-RDSVSRSYDEMFGARSSSFVKPGSVASMFSPAG 117
XptA1       QNKLEELRILSRANPQLANITNLNIT-PSTLNNSYNSWFYGRAHRFVKPGSIASIFSPAA 117
            :*:* * **:.****** .  .* *   .:   .*:. * .*:  :* **:::*:****.

TcdA1       YLTELYREARNLHASDSVYYLDTRRPDLKSMALSQQNMDIELSTLSLSNELLLESIKTES 177
XptA2       YLTELYREAKDLHFSSSAYHLDNRRPDLADLTLSQSNMDTEISTLTLSNELLLEPITRKT 177
XptA1       YLTELYREAKDFHPDNSQYHLNKRRPDIASLALTQNNMDEEISTLSLSNELLLHNIQTLE 177
            *********:::* ..* *:*:.****: .::*:*.*** *:***:*******. *

TcdA1       KLENYTKVMEMLSTFRPSGATPYHDAYENVREVIQLQDPGLEQLNASPAIAGLMHQASLL 237
XptA2       G-GDSDALMESLSTYRQAIDTPYHQPYETIRQVIMTHDSTLSALSRNPEVMGQAEGASLL 236
XptA1       K-TDYNGVMKMLSTYRQTGMTPYHLPYESARQAILLQDKNLTAFSRNTDVAELMDPTSLL 236
               :   :*: ***:* :  ****  **. *:.*  :*  *  :. .  :    . :***

TcdA1       GINASISPELFNILTEEITEGNAEELYKKNFGNI-EPASLAMPEYLKRYYNLSDEELSQF 296
XptA2       AILANISPELYNILTEEITEKNADALFAQNFSENITPENFASQSWIAKYYGLELSEVQKY 296
XptA1       AIKTDISPELYQILVEEITPENSTELMKKNFGTDDV-LIFKSYASLARYYDLSYDELSLF 295
            .* :.*****::**.****  *:  *  :**.       :     : :**.*. .*:. :

TcdA1       IGKASN---FGQQEYSNNQLITPVVNSSDGTVKVYRITREYTTNAYQMD---VELFPFGG 350
XptA2       LGMLQNGYSDSTSAYVDNISTGLVVNN-ESKLEAYKITRVKT-DDYDKHVNYFDLMYEGN 354
XptA1       VNLSF-GKKNTNQQYKNEQLITLVNDG-NDTATARLIKRTRK-DFYDSHLNYAELIPIKE 352
            :.          . * ::     * :. :..  .  *.*  . : *: .    :*:

TcdA1       ENYRLDYKFKNFYNASYLSIKLNDKRELV--RTEGAPQVNIEYSANIT--LNTADISQPF 406
XptA2       NQFFICANFKISREFGATLRKNSGTSGIVG-SLSGPLVANTNFKSNYLSNISDNEYRNGV 413
XptA1       NEYKYNFSVKKTEPDHLDFRLQNGDKEYIYQDKNFVPIANTHY--SIPIKLTTEQITNGI 410
            :::    ..*            ..    :    .    .* .:  .    :.  :  : .

TcdA1       EIGLTRVLPSGSWA-YAAAKFTVEEYNQYSFLLKLNKAIRLSRATELSPTILEGIVRSVN 465
XptA2       KIYAYRYTSSTSATNQGGGIFTFESYPLTIFALKLNKAIRLCLTSGLSPNELQTIVRSDN 473
XptA1       TLRLWRVKPNPSDAINANAHFKMMEFPGDIFLLKLNKAIRLYKATGISPEDIWQVIESIY 470
             :   *   . * :  . . *.. .:    * *********  :: :**  :  ::.*

TcdA1       LQLDINTDVLGKVFLTKYYMQRYAIHAETALILCNAPISQRSYDNQPSQFDRLFNTPLLN 525
XptA2       AQGIINDSVLTKVFYTLFYSHRYALSFDDAQVLNGSVINQYADDDSVSHFNRLFNTPPLK 533
XptA1       DDLTIDSNVLGKLFYVQYYMQHYNISVSDALVLCHSDISQYSTKQQPSHFTMLFNTPLLN 530
             :  *: .** *:* . :* ::* :  . * :*  : *.* : .:. *:*  ***** *:

TcdA1       GQYFSTGDEEIDLNSGS-TGDWRKTILKRAFNIDDVSLFRLLKITDHDNKDGKIKNNLKN 584
XptA2       GKIFEADGNTVSIDPDEEQSTFARSALMRGLGINSGELYQLGKLAGVLDTQNILTLSVPV 593
XptA1       GQEFSADNTKLDLTPGESKNHFYLGIMKRAFRVNDTELYTLWKLANGGTN-PEFMCSIEN 589
            *: *.:..  :.:  ..  . :    : *.: ::. .*: * *::.   .   :  .:

TcdA1       LSNLYIGKLLADIHQLTIDELDLLLIAVGEGKTNLSAISDKQLATLIRKLNTITSWLHTQ 644
XptA2       ISSLYRLTLLARAHQLTVNELCMLYGFSPFNGKTTASLSSGELSRLVIWLYQVTQWLTEA 653
XptA1       LSLLYRVRLLADIHHLTVNELSMLLSVSPYVNTKIALFSDTALTQLISFLFQCTQWLTTQ 649
            :* **   ***  *:**::** :*        .. : :*.  *: *:  *   *.**

TcdA1       KWSVFQLFIMTSTSYNKTLTPEIKNLLDTVYHGLQGFDK---DKADLLHVMAPYIAATLQ 701
XptA2       EITTEAIWLLCTPEFSGNISPEISNLLNTLRPRISEDMAQSSDRELQAEILAPFIAATLH 713
XptA1       KWSVSDVFLMTTDNYSTVLTPDIENLITTLSNGLSTLSL---GDDELIRAAAPLIAASIQ 706
            :  :.  :::: : .:.  ::*:*.**: *:   :.       .     .  ** ***:::

TcdA1       LSSENVAHSVLLWADKLQPGDGAMTAEKFWDWLNTKYTPGSSEAVETQEHIVQYCQALAQ 761
```

```
XptA2      LASPDMARYILLWTDNLRPGGLNIAGFMML-----V--LKETLSDEETTQLVQFCHVMAQ 766
XptA1      MDSAKTAETILLWINQIKPQGLTFDDFMII-----A--ANRDRSENETSNMVAFCQVLGQ 759
           : * . *. :*** ::::* .  :    :              : :   ::* :*:.:.*

TcdA1      LEMVYHSTGINENAFRLFVTKPEMFGAATGAAPAHDALSLIMLTRFADWVNALGEKAS-- 819
XptA2      LSLSVQTLRLSEAELSVLVISDFVVLGARSQ-P----P-------DNTILILCSHSTDST 814
XptA1      LSLIVRNIGLSENELTLLVTKPEKFQSETTA-LQHDLPTLQALTRFHAVIMRCGSYAT-- 816
           *.:  :.  :.*  : ::* .   . .                       :   .  :

TcdA1      SVLAAFEA-----------NSLTAEQLADAMNLDANLLLQASIQAQNHQHLPPVTPENAF 868
XptA2      SGLMGWEIPALTRWICCAKQTLTGDRLG--LRDGAGHQYGN------AG--HGSAGVNQL 864
XptA1      EILTALELG-----------ALTAEQLAVALKFDAQVVTQA------LQ--QTGLGVNTF 857
           . * . *            :**.::*.  :. .*                      * :

TcdA1      SCWTSINTILQWVNVAQQLNVAPQGVSALVGLDYIQSMK----ETPTYAQWENAAGVLTA 924
XptA2      QCWQDINPVLQWIHVASALITMPSVIRTLVNIRYVTALNKAESNLPAWDKWQTLAENMAA 924
XptA1      TNWRTIDVTLQWLDVAATLGITPDGVAALIKLKYVG---EPETPMPTFDDWQAASTLLQA 914
             *  *:  ***:.**  *   *. : :*: : *:          *:: .*:  :  : *

TcdA1      GLNSQQANTLHAFLDESRSAALSTYYIRQVAKAAAAIKSRDDLYQYLLIDNQVSAAIKTT 984
XptA2      GLSTQQAQTLADYTAERLSSVLCNWFLANIQPEGVSLHSRDDLYSYFLIDNQVSSAIKTT 984
XptA1      GLNSQQSDQLQAWLDEATTTAASAYYIKNGAPQ--QIKSRDELYSYLLIDNQVSAQVKTT 972
           **.:**:: *  :  *  ::. . ::: :       ::***:**.*:*******: :***

TcdA1      RIAEAIASIQLYVNRALENVEENANSGVISRQFFIDWDKYNKRYSTWAGVSQLVYYPENY 1044
XptA2      RLAEAIAGIQLYINRALNRIEPNARADVSTRQFFTDWTV-NNRYSTWGGVSRLVYYPENY 1043
XptA1      RVAEAIASIQLYVNRALNNVEGKVSKPVKTRQFFCDWETYNRRYSTWAGVSELAYYPENY 1032
           *:*****.****:****:.:* :.   * :**** **   *.*****.***.*.******

TcdA1      IDPTMRIGQTKMMDALLQSVSQSQLNADTVEDAFMSYLTSFEQVANLKVISAYHDNINND 1104
XptA2      IDPTQRIGQTRMMDELLENISQSQLSRDTVEEAFKTYLTRFETVADLKVVSAYHDNVNSN 1103
XptA1      IDPTIRIGQTGMMNNLLQQLSQSQLNIDTVEDSFKNYLTAFEDVANLQVISGYHDSINVN 1092
           **** ***** **: **:.:*****. ****::* .*** ** **:*:*:*.***.:* :

TcdA1      QGLTYFIGLSETDAGEYYWRSVDHSKFNDGKFAANAWSEWHKIDCPINPYKSTIRPVIYK 1164
XptA2      TGLTWFVGQTRENLPEYYWRNVNISRMQAGELAANAWKEWTKIDTAVNPYKDAIRPVIFR 1163
XptA1      EGLTYLIGYSQTEPRIYYWRNVDHQKCQHGQFAANAWGEWKKIEIPINVWQENIRPVIYK 1152
            ***:::* :. :   ****.*: .: : *::***** ** **:  :* ::. *****::

TcdA1      SRLYLLWLEQKEITKQTGNSKDGYQTETDYRYELKLAHIRYDGTWNTPITFDVNKKISEL 1224
XptA2      ERLHLIWVEKEEVAKNGTDPVET-----YDRFTLKLAFLRHDGSWSAPWSYDITTQVEAV 1218
XptA1      SRLYLLWLEQKELKNESEDGKID-----ITDYILKLSHIRYDGSWSSPFNFNVTDKIENL 1207
           .**:*:*:*::*: ::  :             : ***:.:*:**:*.:* .::. ::. :

TcdA1      KLEKNRAPGLYC---AGYQGEDTLLVMFYNQQDTLDSYK--NASMQGLYIFADMASKDMT 1279
XptA2      TDKK-PDTERLALAASGFQGEDTLLVFVYKTGVSYPDFGDNNKNVAGMTIYGDGSFKKME 1277
XptA1      INKK-ASIGMYC--SSDY-EKDVIIVYFHEKKDNYS-FN-SLPAREGMTINPDMTLSILT 1261
             :*        .   :.:  :*.::* .::   .   :        *: *  * : . :

TcdA1      PEQSNVYRDNS--YQ---QFDTNNVRRVNNRYAEDYEIPSSVSSRKDYGWGDYYLSMVYN 1334
XptA2      NTALSRYSQLKNTFDIIHTQGNDLVRKASYRFAQDFEVPASLNMGS--AIGDDSLTVMEN 1335
XptA1      ENDLDA---------IVKSTLSELDTRTEYKVNNQFATDYLAEYKE--SI---------- 1300
               .                .:   :.. :  :::      .  .  .

TcdA1      GDIPTINYKAASSDLKIYI----------SPKLRIIHNGYE------GQKRNQCNLMNKY 1378
XptA2      GNIPQITSKYSSDNLAITLHNAAFTVRYDGSGNVIRNKQIS-AMKLTGVDG-----KSQY 1389
XptA1      ------TTK----NKSSQFYRNIFDLSYISPGNGHINLTFNPSMEINFSKGNIYNDEVKY 1350
                 . *    :    :           .      :   .         .        :*

TcdA1      GK--LGDKFIVYTSLGV--NPNNSSNKLMFYPVYQYSG------------------NTSG 1416
XptA2      G------------------NAFIIANTVKHYGGYSDLGGPITVYNKTKNYIASVQGHLMN 1431
XptA1      LLSMVEDETVILFDYDRHDEMLGKEEEVFHYGTLD--------------FIIS--IDLKN 1394
                              :     : : .*   .                       .  .

TcdA1      LNQGRLLFHRD--TTYPSKVEAWIPGAKRSLTNQNAAIGDDYATDSLNKP---------- 1464
```

```
XptA2      ADYTRRLILT--------------------------PVENNYYARLFEFPFSPNTILNTV 1465
XptA1      AEYFRVLMHLRTKEKIPRKSEI------------GVGINYDYESDDAEFKLDTNIVLDWK 1442
            :  * *:                             :  :* :   :

TcdA1      ------------------------------DDLKQYIFMTDS---K----GTATDVSGPV 1487
XptA2      FTVGSNK-------------------T---SDFKKCSYAVDGNNSQGFQIFSSYQSSGWL 1503
XptA1      DNTGVWHTICESFTNDVSIINNMGNIAALFLREDPCVYLCSIATD--IKLASS------- 1493
                                            .   :  .          ::

TcdA1      EINTAISPAKVQIIVKAGG-KEQTFTADKDVSIQPSPSFDEMNYQFNALEIDGSGLNFIN 1546
XptA2      DIDTGINNTDVKITVVAGS-KTHTFTASDHIASLPANSFDAMPYTFKPLEIDASSLAFTN 1562
XptA1      -MIEQIQDKNISFLLKNGSDILVELNAEDHVASKPSHESDPMVYDFNQVKVDIEGYDI-- 1550
            :   *.  .:.: :  *.     :.*...::  *: . * * * *: :::* ..  :

TcdA1      NSASIDVTFTAFAEDGRKLGYESFS-IPV-TLKVSTDNALTLHHNENGAQYMQWQSYRTR 1604
XptA2      NIAPLDIVFETKAKDGRVLG-KIKQTLSVKRVNYNPEDILFLRETHSGAQYMQLGVYRIR 1621
XptA1      ---PLVSEFIIKQPDGGYNDIVIESPIHIKLKSKDTSNVISLHKMPSGTQYMQIGPYRTR 1607
               :   *     **   .    . : :   . . .: : *:.  .*:****   ** *

TcdA1      LNTLFARQLVARATTGIDTILSMETQNIQEPQLGKGFYATFVIPPYNLSTHGDERWFKLY 1664
XptA2      LNTLLASQLVSRANTGIDTILTMETQRLPEPPLGEGFFANFVLPKYDPAEHGDERWFKIH 1681
XptA1      LNTLFSRKLAERANIGIDNVLSMETQNLPEPQLGEGFYATFKLPPYNKEEHGDERWFKIH 1667
           ****:: :*. **. ***.:*:****.: ** **:**:*.* :* *:   ********::

TcdA1      IKHVVDNNSH-IIYSGQLTDT-NINITLFIPLDDVPLNQDYHAKVYMTFKKSPSDGTWWG 1722
XptA2      IGNVGGNTGRQPYYSGMLSDTSETSMTLFVPYAEGYYMH-EGVRLGVGYQKITYDNTWES 1740
XptA1      IGNIDGNSARQPYYEGMLSDI-ETTVTLFVPYAKGYYIR-EGVRLGVGYKKIIYDKSWES 1725
           * :: .*..:   *.* *:*  : .:***:*  .    :   .:: : ::*   * :* .

TcdA1      PHFVRDDKG--IVTINP---------KSIL------THFESVNVNLNNISSEPMDFSGANS 1765
XptA2      AFFYFDETKQQFVLINDADHDSGMTQQGIVKNIKKYKGFLNVS-IATGYSAPMDFNSASA 1799
XptA1      AFFYFDETKNQFIFINDADHDSGMTQQGIVKNIKKYKGFIHVV-VMKNNTEPMDFNGANA 1784
            .*  *:.   :: **          :.*:      . *  *  : .  : ****..*.:

TcdA1      LYFWELFYYTPMLVAQRLLHEQNFDEANRWLKYVWSPSGYIVHGQIQNYQWNVRPLLEDT 1825
XptA2      LYYWELFYYTPMMCFQRLLQEKQFDEATQWINYVYNPAGYIVNGEIAPWIWNCRPLEETT 1859
XptA1      IYFWELFYYTPMMVFQRLLQEQNFTESTRWLRYIWNPAGYSVQGEMQDYYWNVRPLEEDT 1844
           :*:*********:  ****:*::* *:.:*:.*::.*:** *:*::  : ** *** * *

TcdA1      SWNSDPLDSVDPDAVAQHDPMHYKVSTFMRTLDLLIARGDHAYRQLERDTLNEAKMWYMQ 1885
XptA2      SWNANPLDAIDPDAVAQYDPTHYKVATFMRLLDQLILRGDMAYRELTRDALNEAKMWYVR 1919
XptA1      SWNANPLDSVDPDAVAQHDPMHYKVATFMKMLDLLITRGDSAYRQLERDTLNEAKMWYVQ 1904
           ***::***::*******:** ****:***: ** ** *** ***:* **:********::

TcdA1      ALHLLGDKPYLPLSTTWSDPRLDRAADITTQNAHDSAIVALRQNIPTPAPLSLRSANTLT 1945
XptA2      ALELLGDEPEDYGSQQWAAPSLSVAASHTVQAAYQQDLTALDNGEVATQ---PRTANSLV 1976
XptA1      ALTLLGDEPYFSLDNDWSEPRLEEAASQTMRHHYQHKMLQLRQRAALPT---KRTANSLT 1961
           ** ****:*    .  *: * *. **. * :  ::  :  * :          *:**:*.

TcdA1      DLFLPQINEVMMNYWQTLAQRVYNLRHNLSIDGQPLYLPIYATPADPKALLSAAVATSQG 2005
XptA2      GLFLPEYNPALTDYWQTLRLRLFNLRHNLSIDGQPLSLAIYAEPTDPKALLPSMVQASQG 2036
XptA1      ALFLPQINKKLQGYWQTLTQRLYNLRHNLTIDGQPLSLSLYATPADPSMLLSAAITASQG 2021
            ****: *  : .*****  *::******:****** * :** *:**. ** : : :***

TcdA1      GGKLPESFMSLWRFPHMLENARGMVSQLTQFGSTLQNIIERQDAEALNALLQNQAAELIL 2065
XptA2      GSAVLPGTLSLYRFPVMLERARNLVAQLTQFGTSLLSMAEHDDADELTTLLLQQGMELAT 2096
XptA1      GGDLPHAVMPMYRFPVILENAKWGVSQLIQFGNTLLSITERQDAEALAEILQTQGSELAL 2081
           *. :  . : ::*** :**.*:  *:** ***.:* .: *::**: *  :*  *. **

TcdA1      TNLSIQDKTIEELDAEKTVLEKSKAGAQSRFDSYGKLYDENINAGENQAMTLRASAAGLT 2125
XptA2      QSIRIQQRTVDEVDADIAVLAESRRSAQNRLEKYQQLYDEDINHGEQRAMSLFDAAAGQS 2156
XptA1      QSIKMQDKVMAEIDADKLALQESRHGAQSRFDSFNTLYDEDVNAGEKQAMDLYLSSSVLS 2141
            .: :*::.: *:**:  .* :*: .**.*::.:  ****::* **::** *  :::  :

TcdA1      TAVQASRLAGAAADLVPNIFGFAGGGSRWGAIAEATGYVMEFSANVMNTEADKISQSETY 2185
```

```
XptA2      LAGQALSVAEGVADLVPNVFGFACGGSRWGAALRASASVMSLSATASQYSADKISRSEAY 2216
XptA1      TSGTALHMAAAAADLVPNIYGFAVGGSRFGALFNASAIGIEISASATRIAADKISQSEIY 2201
            :  *  :* ..******::*** ****:**  .*:.  :.:**.. .  *****:** *

TcdA1      RRRRQEWEIQRNNAEAELKQIDAQLKSLAVRREAAVLQKTSLKTQQEQTQSQLAFLQRKF 2245
XptA2      RRRRQEWEIQRDNADGEVKQMDAQLESLKIRREAAQMQVEYQETQQAHTQAQLELLQRKF 2276
XptA1      RRRRQEWEIQRNNAEAEIKQIDAQLATLAVRREAAVLQKNYLETQQAQTQAQLAFLQSKF 2261
           ***********:**:.*:**:**** :* :***** :*    :*** :**:** :** **

TcdA1      SNQALYNWLRGRLAAIYFQFYDLAVARCLMAEQAYRWELNDDSARFIKPGAWQGTYAGLL 2305
XptA2      TNKALYSWMRGKLSAIYYQFFDLTQSFCLMAQEALRRELTDNGVTFIRGGAWNGTTAGLM 2336
XptA1      SNAALYNWLRGRLSAIYYQFYDLAVSLCLMAEQTYQYELNNAAAHFIKPGAWHGTYAGLL 2321
           :* ***.*:**:*:***:**:**: : ****::: : **.: .. **: ***:** ***:

TcdA1      AGETLMLSLAQMEDAHLKRDKRALEVERTVSLAEVYAGLPKDNGPFSLAQEIDKLVSQGS 2365
XptA2      AGETLLLNLAEMEKVWLERDERALEVTRTVSLAQFYQALSSD--NFNLTEKLTQFLREGK 2394
XptA1      AGETLMLNLAQMEKSYLEKDERALEVTRTVSLAEVYAGLTEN--SFILKDKVTELVNAGE 2379
           *****:*.**:**.  *::*:***** ******:.* .* .:   * * ::: :::  *.

TcdA1      GSAGSGNNNLAFGAGTDTKTSLQASVSFADLKIREDYPASLGKIRRIKQISVTLPALLGP 2425
XptA2      GNVGASGNELKLS-----NRQIEASVRLSDLKIFSDYPESFGNTRQLKQVSVTLPALVGP 2449
XptA1      GSAGTTLNGLNVE-----GTQLQASLKLSDLNIATDYPDGLGNTRRIKQISVTLPALLGP 2434
           *..*:  * * .        .::**: ::**:*  *** .:*: *::**:*******:**

TcdA1      YQDVQAILSYGDKAGLANGCEALAVSHGMNDSGQFQLDFNDGKFLPFEGIAI-DQGTLTL 2484
XptA2      YEDIRAVLNYGGSIVMPRGCSAIALSHGVNDSGQFMLDFNDSRYLPFEGISVNDSGSLTL 2509
XptA1      YQDVRAILSYGGSTMMPRGCKAIVISHGMNDSGQFQMDFNDAKYLPFEGLPVADTGTLTL 2494
           *:*::*:*.**..  : .**.*:.:***:****** :****.::*****: : * *:***

TcdA1      SFPNASMPEKGKQATMLKTLNDIILHIRYTIK-    2516
XptA2      SFPDAT----DRQKALLESLSDIILHIRYTIRS    2538
XptA1      SFPGIS----GKQKSLLLSLSDIILHIRYTIRS    2523
           ***. :    .:* ::* :*.**********:
```

Figure S1. Sequence alignment between *Photorhabdus luminescens* TcdA1 *Xenorhabdus nematophilus* XptA2 and XptA1. Asterisks (*) denote residues between all three proteins that are identical, two dots indicate high similarity, and one dot indicates moderate similarity. XptA2 shares ~43% sequence identity with TcdA1 and ~46% sequence identity with XptA1. Alignments were created using EMBL-EBI Clustal Omega Multiple Sequence Alignment program.

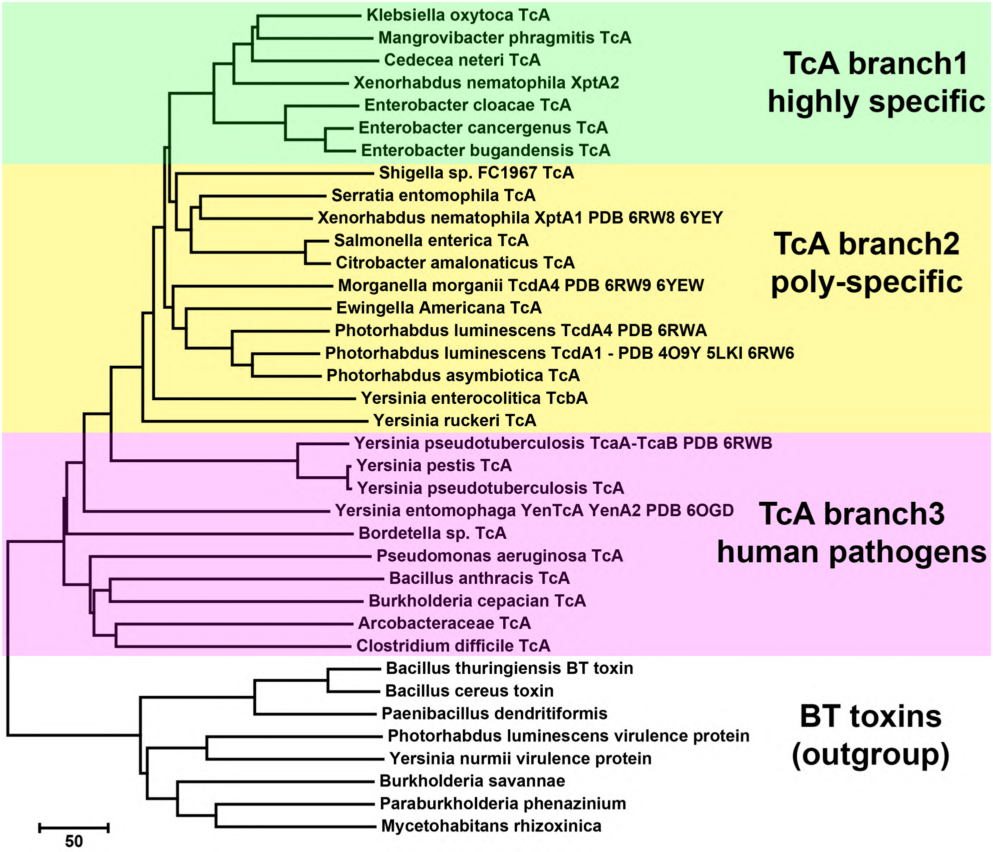

Figure S2. Phylogenetic relationships of bacterial toxin complexes. Evolutionary history is inferred using the Neighbor-Joining method (1). The optimal tree is drawn to scale, with branch lengths in the same units as those of the evolutionary distances used to infer the tree. Distances were calculated using the Poisson correction method (2) and are in the units of the number of amino acids substitutions per site. The percentage of replicate trees in which the associated taxa clustered together in the bootstrap test using 500 replicates (3) are shown at selected branchpoints. Analyses were conducted using MEGA11 (4) using only full-length sequences for all toxins.

**PDB**	8TV0
**Data collection**	
Beamline	APS 22-ID
Wavelength (λ)	0.979 (Se K-edge)
Resolution range (Å) [a]	48.8 – 3.10 (3.21 – 3.10)
Space group	P 21 21 21
Unit cell (a,b,c,α,β,γ)	175.1 176.9 509.7 90 90 90
Total reflections[a]	894,432
Unique reflections[a]	271,071 (19,970)
Multiplicity[a]	3.3 (1.9)
Completeness (%)[a]	94.73 (70.51)
Mean I/σ(I) [a]	30.9 (6.69)
Wilson B-factor (Å^2)	55.84
R_{merge}[a]	0.090 (0.149)
R_{meas}[a]	0.106 (0.197)
R_{pim}[a]	0.055 (0.127)
$CC_{1/2}$[a]	0.95
CC*[a]	0.987
**Refinement**	
Reflections used in	271,034 (19,970)
Reflections used for R-	1829 (130)
R_{work}[a]	0.1940 (0.2794)
R_{free}[a,b]	0.2454 (0.3420)
Number of non-H atoms	100,035
macromolecules	100,035
Ligands (metal ions)	0
solvent	0
Protein residues	12,685
R.m.s.d. (Å, bonds)	0.002
R.m.s.d. (˚, angles)	0.52
Ramachandran favored	95.01
Ramachandran allowed	4.68
Ramachandran outliers	0.31
Rotamer outliers (%)	0.03
Clashscore	5.41
Average B-factor (Å^2)	49.8

Table S1. Crystallographic data and refinement statistics.

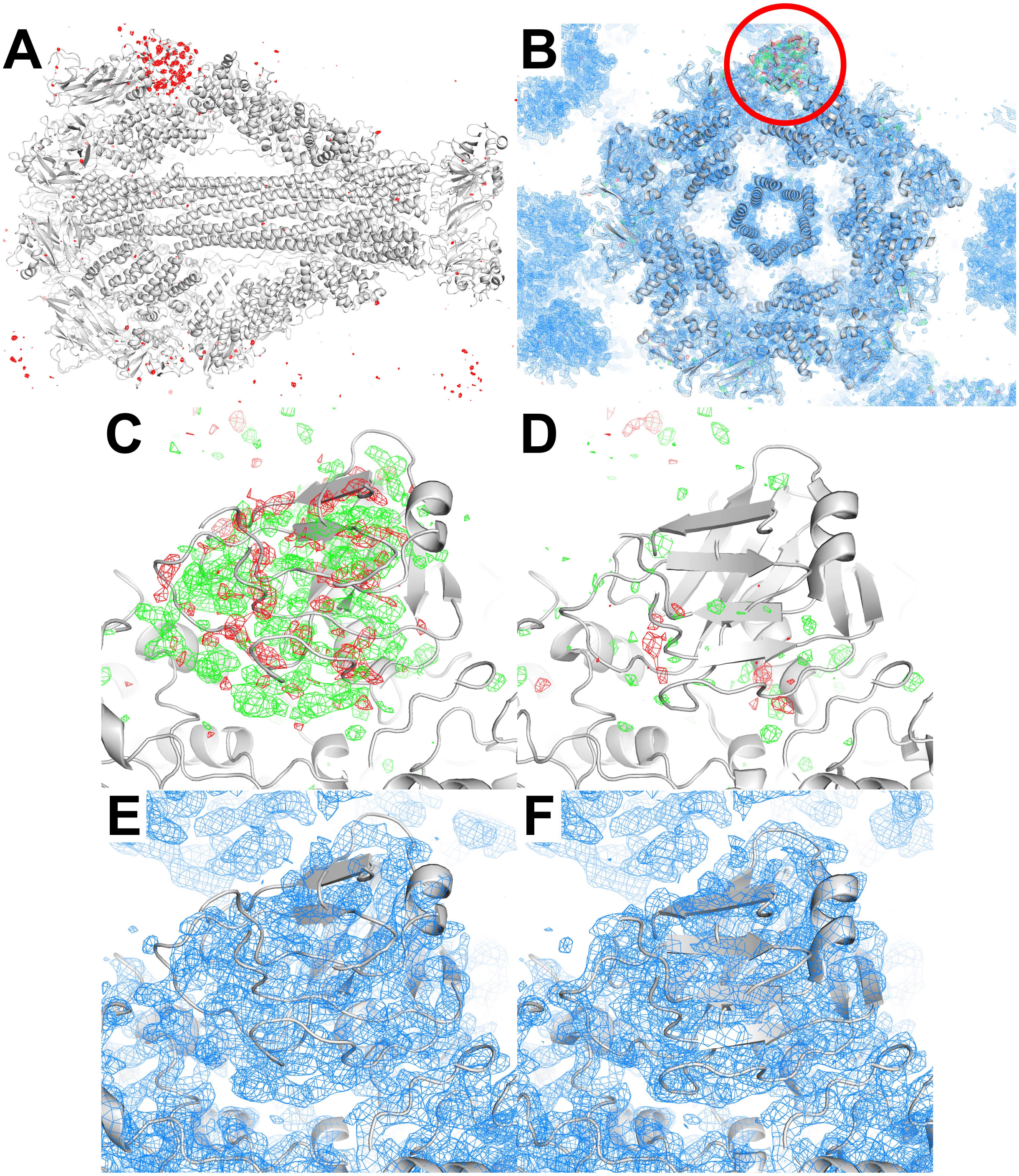

Figure S3. Special NCS refinement of XptA2, RBD-B and electron density maps. The atomic model (white cartoon) for the XptA2 pentamer was refined against the x-ray diffraction data using full NCS, i.e. all atoms for all five chains, which is equivalent to C5 symmetry in cryo-EM (panels A, B, C and E). Panels D and F show the refined model (also white cartoon) with special NCS that does not constrain RBD-B specifically for any of the five chains. Closeup of RBD-B is shown in panels C, D, E and F. 2mFo-DFc density is shown in blue mesh for panels B, E and F (1 σ contour). Positively contoured electron density (+3.5 σ) is shown in green mesh for panels B, C and D. Negatively contoured electron density (-4 σ) is shown in red mesh for panels A, B, C and D. Red circle highlights RBD-B in panel B.

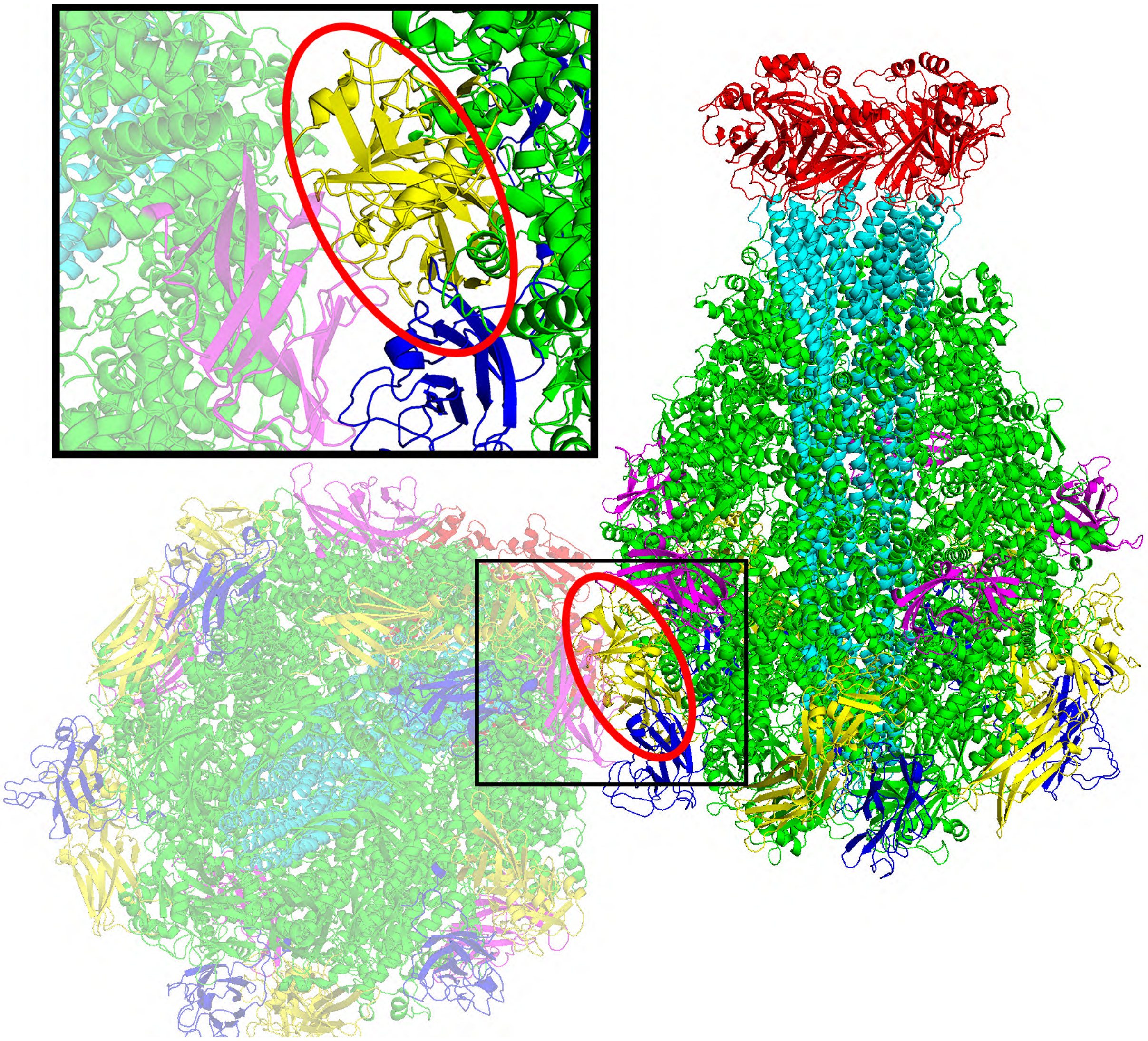

Figure S4. Crystal lattice packing of XptA2 at the RBD-B interface. Two XptA2 molecules in the crystal lattice pack against each other (small square outline) at an interface (small red circular outline) formed by RBD-B of one molecule (yellow) and RBD-A of another molecule (magenta). Color coding of the domains is the same as in Figure 2 of the main manuscript. Inset shows a zoom with a slight rotation to enhance the view of the RBD-B interface. Figure 3 of the main manuscript shows the effect of crystal packing on RBD-B when the crystal structure is compared to the cryo-EM structure.

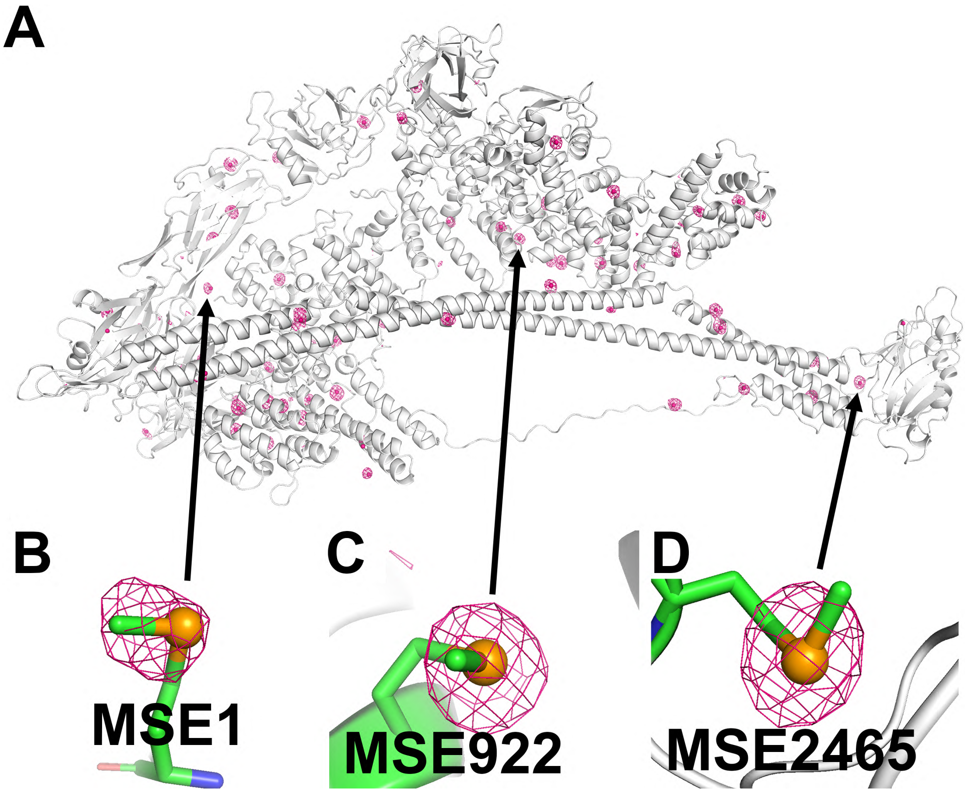

Figure S5. X-ray crystal structure of Seleno-methionine (Se-Met) incorporated XptA2. Anomalous difference fourier electron density is shown in red mesh, contoured to 4 σ, and overlaid with the refined atomic model of XptA2 in all panels.

(A) Chain A of XptA2 depicted in white cartoon. 58 of 61 total Se-Met per chain (i.e. ~93%) exhibit strong associated anomalous difference fourier electron density.

(B) Zoom for the first residue of XptA2, the start codon SeMet1, designated “MSE1”.

(C) Zoom for a SeMet located midway through the polypeptide, “MSE922”.

(D) Zoom for a SeMet located near the C-terminus of XptA2, “MSE2465.

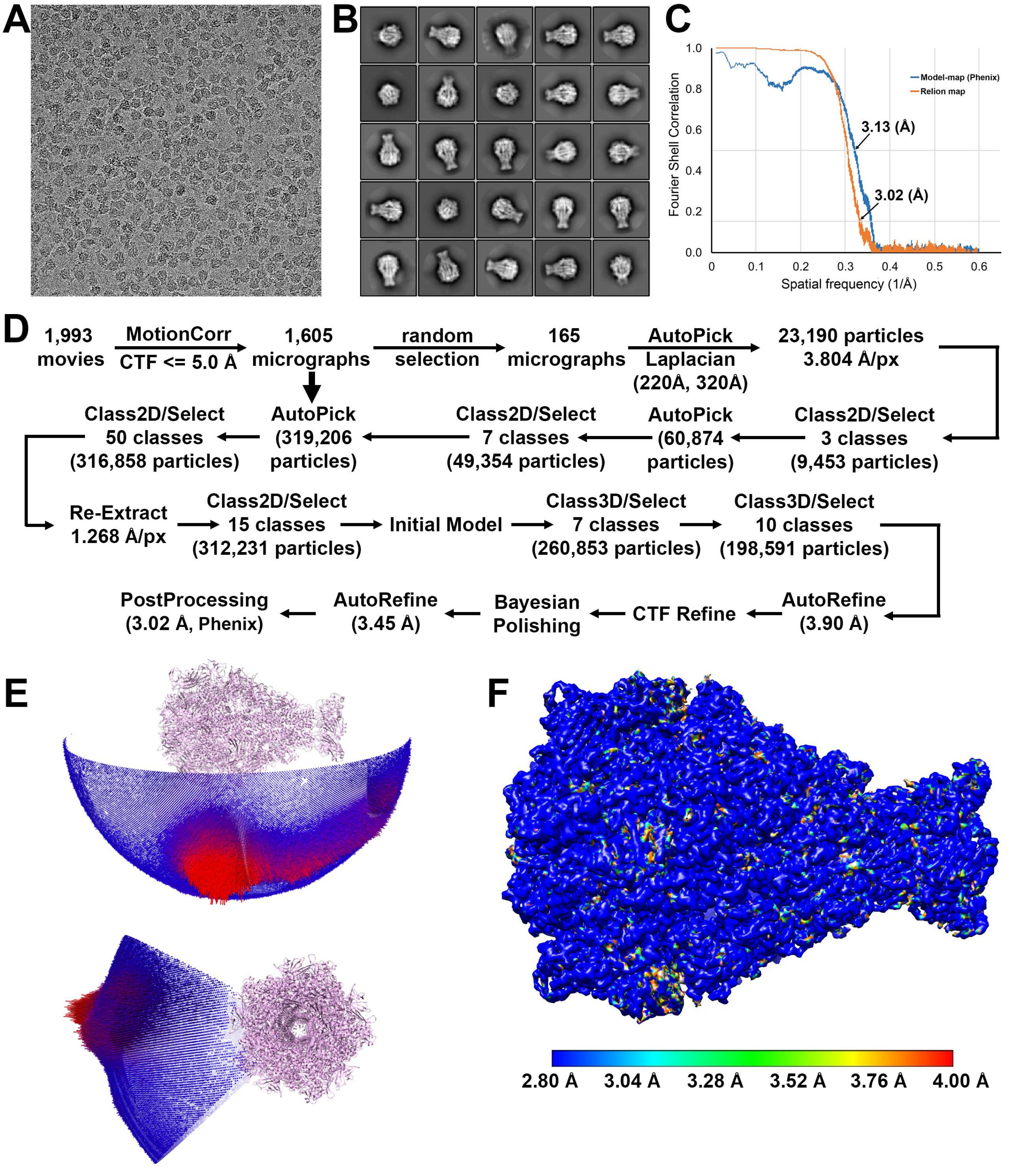

Figure S6. Cryo-EM processing and analysis of XptA2.
(A) Representative cryo-EM micrograph.
(B) Representative cryo-EM 2D class averages.
(C) FSC curves of the refined model (blue) versus the overall 3.1 Å map that it was refined against (orange).
(D) A flowchart for the cryo-EM data processing and structure determination of the *Xenorhabdus nematophilus* XptA2.
(E) Euler angle distribution of the final 3D refinement of the overall map.
(F). Local resolution map calculated using RELION 3.0.8 (5).

	XptA2 pre-pore (PDB code 8TQE)	XptA2 pre-pore "2frag"
Microscope	FEI Polara	Technai T20
Voltage (kV)	300	200
Camera	K2	K2
Energy filter	none	none
Physical pixel size	1.268	0.649
Defocus range (µm)	-0.5 to -2.5	-0.5 to -2.5
**Cryo-EM Data and Map**		
Number of micrographs	1605	26
Number of particles (3D refinement)	198,591	3,477
Resolution (dFSC half maps; 0.143, Å)	3.02	8.3
Sharpening B-factor (Å²)	-107	-150
**Model vs. Map**		
CC (mask)	0.86	0.79
CC (peak)	0.79	0.72
CC (volume)	0.84	0.76
d FSC model (0.5, Å)	3.1	8.9
**Model**		
Number of chains	5	5
Number of protein atoms	12685	12685
RMSD bond length (Å)	0.004	0.019
RMSD bond angles	0.667	1.157
Molprobity Score	1.62	2.13
All atom clash score	5.23	21.48
Ramachandran Favored (%)	95.05	95.57
Ramachandran Allowed (%)	4.69	4.2
Ramachandran Outliers (%)	0.27	0.24
Rotamer Outliers (%)	0.19	0
Cβ outliers (%)	0	0.21

Table S2. Cryo-EM data collection and refinement statistics for both the full-length wild type XptA2 and the “2-fragment” XptA2 construct.

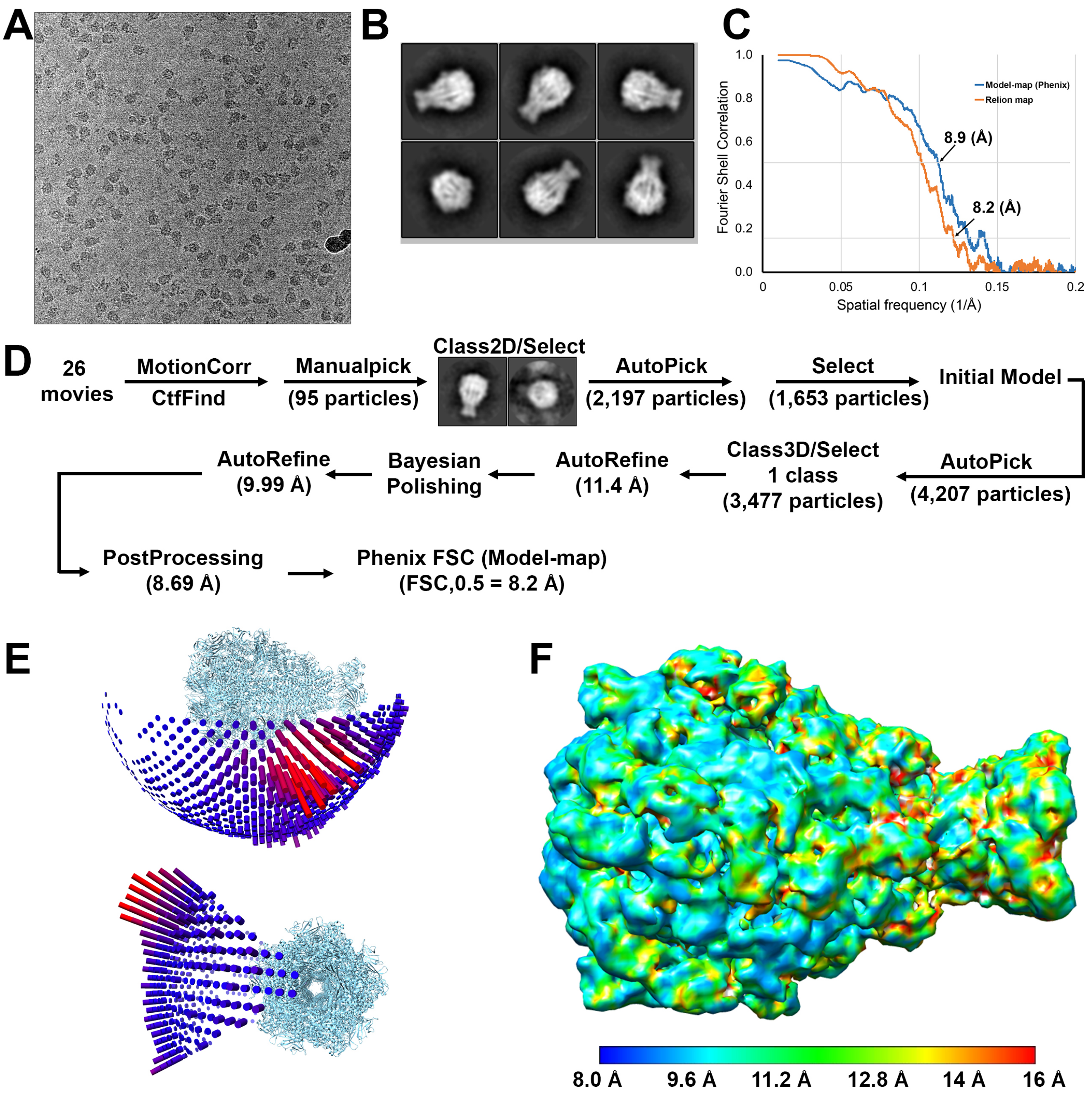

Figure S7. Cryo-EM processing and analysis of the XptA2 2-fragment construct.
(A) Representative cryo-EM micrograph.
(B) Representative cryo-EM 2D class averages.
(C) FSC curves of the refined model (blue) versus the overall 8.2 Å map that it was refined against (orange).
(D) A flowchart for the cryo-EM data processing and structure determination of the *Xenorhabdus nematophilus* XptA2 2-fragment construct.
(E) Euler angle distribution of the final 3D refinement of the overall map. F. Local resolution map calculated using RELION 3.0.

References used in this supplement

1. Saitou N, Nei M. The neighbor-joining method: a new method for reconstructing phylogenetic trees. Mol Biol Evol. 1987;4(4):406-25. doi: 10.1093/oxfordjournals.molbev.a040454. PubMed PMID: 3447015.
2. Zuckerkandl E. Evolutionary divergence and convergence in proteins: Academic Press, New York; 1965.
3. Felsenstein J. Confidence Limits on Phylogenies: An Approach Using the Bootstrap. Evolution. 1985;39(4):783-91. doi: 10.1111/j.1558-5646.1985.tb00420.x. PubMed PMID: 28561359.
4. Tamura K, Stecher G, Kumar S. MEGA11: Molecular Evolutionary Genetics Analysis Version 11. Mol Biol Evol. 2021;38(7):3022-7. doi: 10.1093/molbev/msab120. PubMed PMID: 33892491; PMCID: PMC8233496.
5. Zivanov J, Nakane T, Scheres SHW. Estimation of high-order aberrations and anisotropic magnification from cryo-EM data sets in RELION-3.1. IUCrJ. 2020;7(Pt 2):253-67. Epub 20200211. doi: 10.1107/S2052252520000081. PubMed PMID: 32148853; PMCID: PMC7055373.
